# Survival After Treatable Hepatocellular Carcinoma Recurrence in Liver Recipients: A Nationwide Cohort Analysis

**DOI:** 10.3389/fonc.2020.616094

**Published:** 2021-01-28

**Authors:** Cheng-Maw Ho, Chih-Hsin Lee, Ming-Chia Lee, Jun-Fu Zhang, Chin-Hua Chen, Jann-Yuan Wang, Rey-Heng Hu, Po-Huang Lee

**Affiliations:** ^1^Department of Surgery, National Taiwan University Hospital and College of Medicine, Taipei, Taiwan; ^2^Division of Pulmonary Medicine and Pulmonary Research Center, Wanfang Hospital, Taipei Medical University, Taipei, Taiwan; ^3^Department of Pharmacy, New Taipei City Hospital, New Taipei City, Taiwan; ^4^School of Pharmacy, College of Pharmacy, Taipei Medical University, Taipei, Taiwan; ^5^Biostatistics Center, College of Management, Taipei Medical University, Taipei, Taiwan; ^6^Big Data Center, Lo-Hsu Medical Foundation, Lotung Poh-Ai Hospital, Yilan, Taiwan; ^7^Graduate Institute of Data Science, College of Management, Taipei Medical University, Taipei, Taiwan; ^8^Department of Medical Education and Research, Wanfang Hospital, Taipei Medical University, Taipei, Taiwan; ^9^Department of Internal Medicine, National Taiwan University Hospital, and College of Medicine, Taipei, Taiwan

**Keywords:** hepatocellular carcinoma, liver transplantation, radiofrequency ablation, recurrence, survival

## Abstract

**Background:**

Survival after post-transplant recurrence of HCC is dismal, and almost all treatments for recurrent HCC are off-labeled, without an extensive large-scale analysis. We aimed to delineate their post-recurrence courses and define benchmarks for comparing future treatment effectiveness.

**Methods:**

Three national databases, including health insurance, catastrophic illness, and the cause of death, were linked for cohort establishment and data collection during the period from 2005 to 2016. Patients with HCC recurrence ≥6 months after transplant surgery and under treatment were recruited for survival analysis. Selection of treatment strategies for HCC recurrence after liver transplant was based on the same criteria for those without liver transplant.

**Results:**

Of 2,123 liver transplant recipients, 349 developed HCC recurrence ≥6 months after liver transplant, and the median recurrence time was 17.8 months post-transplant. Within 2 years of treatment, 61% patients showed recurrence (early recurrence group), and survival in these patients was poorer than in the late recurrence group. According to a multivariable analysis, the transplant era before 2008 and radiofrequency ablation were associated with good prognosis, whereas receiving sorafenib and radiotherapy was associated with poor prognosis. The effect of transplant era became insignificant after stratification by recently receiving pretransplant transarterial chemoembolization.

**Conclusion:**

Timing of recurrence and interventions used were associated with the outcomes of patients with post-transplant HCC recurrence. These data provide the benchmark and indicate the critical period and high-risk factors for further therapeutic trial consideration.

## Introduction

Patients with HCC have high recurrence rates after cancer treatment (1). Although primary HCC can be cured through liver transplant under stringent criteria (2), the current trend of accommodating transplant patients through relaxing criteria and salvaging those who had recurrent HCC with multiple previous loco-regional treatments can potentially increase the pool of post-transplant recurrence tremendously in the near future (1, 3). However, guidelines for the management of HCC recurrence after liver transplantation are still lacking (3).

Currently, the management strategy of primary HCC and non-transplant setting is used for post-transplant HCC recurrence (3, 4). Thriving clinical trials on newer systemic therapies, such as target therapy and immunotherapy, which can prolong patient survival after recurrence, have always excluded transplant patients (5). Consequently, almost all transplant patients with HCC recurrence were neglected and received off-labeled cancer treatments. With the changing landscape of HCC and the approval of new systemic chemotherapeutic agents, future studies are warranted to characterize the efficacy and safety of these agents in liver transplant recipients (3).

Numerous studies have emphasized on the primary prevention of HCC recurrence (or re-recurrence) after liver transplantation (6–13) rather than on prolonging meaningful outcomes after recurrence. To address this emerging critical issue, large-scale studies are necessary but remain scant (14–16). Without a benchmark reference, institutional bias and limited overview exist in this heterogeneous population.

HCC is one of the leading causes of cancer-related mortality in Taiwan for decades, and liver transplantation is a mature surgery performed in nationwide multiple centers (17). With a longitudinal follow-up of more than 20 million patients and validated diagnoses of catastrophic illnesses, the National Health Insurance Research Database (NHIRD) provides a great platform to explore the clinical course and outcome of post-transplant recurrence.

Particularly, we aimed to illustrate the courses of post-transplant HCC recurrence by using the NHIRD as a source material and to analyze relevant prognostic factors. Additionally, this study enriches the literature and provides a benchmark reference for comparing effectiveness in future interventional analyses.

## Methods

The Institutional Review Board of National Taiwan University Hospital, Taipei, Taiwan, approved this study (NTUH REC: 201601007W). Because this was a retrospective study using an encrypted database, the institutional review board waived the need for informed consent.

### Data Acquisition

Entire original data were from the following three linked national databases covering the beneficiaries of the whole population of Taiwan from 2005 to 2016: Taiwan’s NHIRD, Registry for Catastrophic Illness Patient Database (RCIPD), and Cause of Death Database. Regarding HCC, the histologic confirmation or typical imaging presentation is required for registering patients in the RCIPD.

### Cohort Selection

Patients with HCC who received liver transplant surgery were identified from Taiwan’s NHIRD. Regardless of donor types, liver transplant surgery for HCC is reimbursed if the tumor status listed and at transplant is within the University of California San Francisco criteria (single tumor <6.5 cm, maximum of three total tumors with none >4.5 cm, and cumulative tumor size <8 cm) (1, 18, 19). Image follow-ups (every 3 to 6 months) were regularly performed for detection of recurrence. Among them, those with HCC recurrence were identified. In this study, HCC recurrence was diagnosed as having a compatible diagnostic code (International Classification of Diseases, 9th/10th Revision 155/C22) and receiving intervention, to validate the definite recurrence and to identify patients with treatable diseases. The main cohort adopted liver recipients who had recurrence ≥6 months after transplant surgery as the target population.

The date of the first intervention for treatable HCC recurrence after transplantation was defined as the index date. Interventions included hepatectomy (resection), radiofrequency ablation (RFA), transarterial chemoembolization (TACE), radiotherapy, sorafenib, and chemotherapy. The coding of interventions is detailed in the **Supplement**.

Liver transplant recipients who survived or had HCC recurrence <180 days after transplant surgery were excluded because within this period, high rejection rates, surgical complications, and infection episodes interfere in the appropriate assessment for cancer-related survival. Moreover, adjuvant systemic therapy, such as sorafenib or chemotherapy, may be administered in this period, confounding true HCC recurrence (20).

### Treatment Strategies for Post-Transplant HCC Recurrence

Selection of treatment strategies for HCC recurrence after liver transplant was based on the same criteria for those without liver transplant (17, 21). Particularly for post-transplant recurrence, extensive tumor staging would be performed initially to identify the intra- and extra-hepatic involvements before treatments. Systemic therapy with sorafenib was used in patients with vascular or extrahepatic metastases. Locoregional therapies (resection, RFA, or TACE) were performed for intra-hepatic recurrence with curative intents (resection and RFA) as the priority consideration.

Since 2011, treatment decisions for patients with HCC awaiting liver transplant were audited at each center’s multidisciplinary liver tumor board, attended by hepatologists, liver transplant surgeons, oncologists, and radiologists with an expertise in HCC management.

### Pre-Claim Review of High-Priced Interventions

In Taiwan, liver transplant surgery, RFA, and sorafenib are regulated clinical treatments that need a pre-claim review process of charts and images before being implemented and reimbursed. RFA for HCC, confined within liver, is approved if the tumor number is not more than three and each tumor size is <5 cm in diameter. Sorafenib for HCC is approved if the patient has distant metastases or major vascular invasion inside the well-reserved liver.

### Demographic Parameters

Demographic information, namely sex, age, monthly income, transplantation period, liver cirrhosis, and underlying comorbidity (such as diabetes mellitus, hyperlipidemia, and alcohol use), was collected, as described previously (22). Viral status, including HBV or HCV, was defined based on the prescription of antiviral medications, at least two outpatient coding, or at least one inpatient coding of the corresponding viral diagnosis within 1 year before transplantation. Reimbursement of direct-acting antiviral agents for HCV in Taiwan started since January 2017, beyond the study period (December 2016), and was therefore not included in analysis. The details of coding definitions are described in the **Supplement**.

When more than one treatment modality was used as the initial treatment (such as TACE and RFA at the same admission), the one with curative intent (resection or RFA) was prioritized and coded. Furthermore, interventions for HCC within 1 year before liver transplant surgery were collected. A minimum observation period of 3 months between local treatment and transplantation is a consensus policy required for successful downstaging of HCC (23).

Living liver donation was coded when the period of living donor surgery (procedural code: 75022B) and hospital stay overlapped with the period of liver transplant surgery and hospital stay of the recipient in the same hospital. Deceased liver donation, after brain death as only allowed by law in the studied period, was coded when liver transplant surgery and recipients’ hospital stay overlapped with the date of deceased liver donation surgery (75021B).

The doses of post-transplant medications, including HBV medications (lamivudine, entecavir, and tenofovir), metformin, and immunosuppressants (tacrolimus [anatomical therapeutic chemical (ATC) code: L04AD02], cyclosporin [ATC code: L04AD01], mycophenolate mofetil [MMF]/myfortic acid [ATC code: L04AA06], sirolimus [ATC code: L04AA10], and everolimus [ATC code: L04AA18]), within 180 days after transplant surgery were calculated. Drug codes other than immunosuppressants were described previously (24).

### Outcome Measurement

The patients were followed up until death, withdrawal of health insurance, or December 31, 2016. The event date was the date of death or the last follow-up date. The date of death was obtained from the Cause of Death Database. Death due to HCC was considered when the first two diagnoses on the death certificate included HCC. Overall survival and HCC-specific survival were estimated separately.

### Statistical Analysis

A recent review suggested that HCC recurring >2 years after liver transplantation may host a different biological mechanism compared with early recurrence (3). Therefore, in our study, patients were stratified into two groups based on the time to recurrence, that is, recurrence within 2 years after transplantation and recurrence beyond 2 years after transplantation. In the 12-year study period, three eras (2005–2008, 2009–2012, and 2013–2016) were split even for historical comparison. Liver recipients with treatable HCC recurrence within 6 months after transplant surgery and patients with recurrence code but received no treatment were processed separately for assessment of data quality and plausibility.

Data are expressed as the mean ± standard deviation, median (interquartile range [IQR]), or number (percentage) as appropriate. Student’s t test or χ² test was used for the intergroup comparison. The time-to-event curves were generated using the Kaplan–Meier method and compared using the log-rank test.

Cox’s proportional hazard model was used for univariable and multivariable analyses. Sensitivity analysis was performed for a cohort of recipients who could be matched to one single living or deceased donor and for a subgroup of patients with primary HCC treated by upfront transplant. All statistical tests were two-sided at a significance level of 0.05, and all analyses were performed using SAS, version 9.4 (SAS Institute Inc., Cary, NC, USA).

## Results

### Demographics

**Figure 1:**
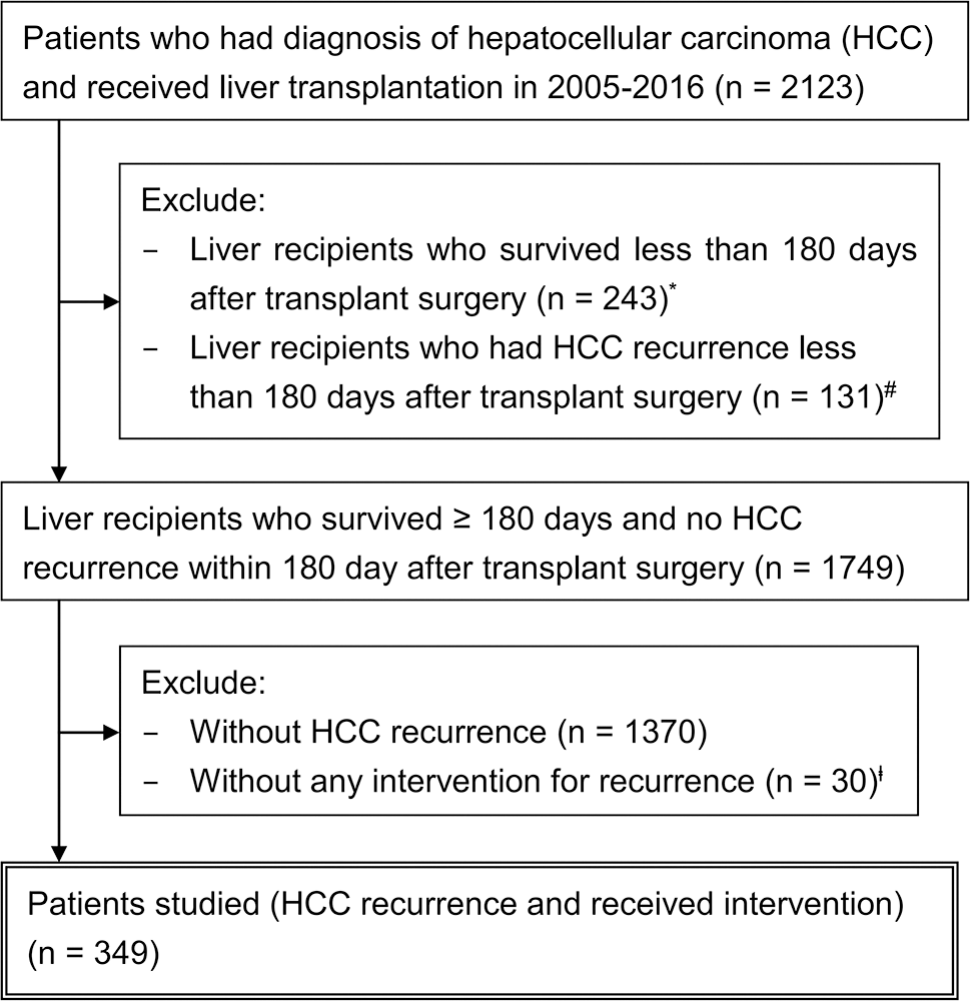
Schematic representation of the patient selection process. *Contained patients with HCC recurrence deemed unfit or too advanced for any treatment. #Within 6 months after transplant surgery, 41 (31.3%) patients had complications (rejection, surgical complication, or sepsis) and 21 (16.0%) expired. The initial interventions included radiotherapy (n = 37), chemotherapy (n = 35), sorafenib (n = 26), transarterial chemoembolization (n = 25), and others (n = 8). However, 33 patients (25.2%) survived unusually longer than 5 years, suggesting data miscoding or adjuvant treatments, rather than true recurrence. ╪14 (46.7%) patients had complications within 6 months after transplant surgery. Sixteen patients (53.3%) survived unusually longer than 5 years without any oncological interventions, suggesting another area of miscoding and data contamination. These patients (# and ╪) were excluded from the main cohort for analysis because their data were highly heterogeneous and lack of confidence, and validated discrimination between true recurrence and false positivity was not possible.

From the RCIPD and NHIRD, 2,123 patients who had HCC diagnosis and received liver transplantation in 2005–2016 were identified (**Figure 1**). The calculated post-transplant HCC recurrence rate was 24.0% (510/2123). Among them, 349 patients who developed HCC recurrence >6 months after liver transplant and were undergoing treatment were included in this study, excluding 131 patients who claimed to have recurrence within 6 months after transplantation and 30 patients who did not receive any intervention (marked in **Figure 1**).

**Table 1** shows the characteristics of this cohort, which was composed of patients with an average age of 55.4 years; furthermore, 84.5% of patients were men; 90.0, 75.1, 43.0, and 22.6% had cirrhosis, HBV exposure, HCV exposure, and diabetes mellitus, respectively; and 76.2% had received living donor liver transplantation. Among 150 patients with HCV-related HCC (97 co-infected with HBV), 39 (26.0%) received pre-transplant, and 14 (9.3%) post-transplant, anti-HCV therapy. The median follow-up duration after transplantation was 33.9 months (IQR, 20.7–61.4 months) in the cohort, 24.7 months (16.6–32.6 months) in the early recurrence group, and 65.3 months (47.9–88.5 months) in the late recurrence group (*P* < 0.001). The median interval between transplant and post-transplant recurrence was 17.8 months (10.7–34.4 months), 11.4 months (8.3–16.5 months) in the early recurrence group, and 39.8 months (30.6–60.3 months) in the late recurrence group (*P* < 0.001). The distribution of patients among the three transplant eras was statistically different (*P* < 0.001) in terms of early and late recurrence. After 2013, nearly 41% and only 11.8% of patients in the early and late recurrence groups, respectively, received transplant surgery.

**Table 1:** Patient demographics.

	All recurrence (n = 349)	Recur within 2 years (n = 213)	Recur after 2 years (n = 136)	*P*-value
Survival after recurrence: months (median, IQR)	11.2 (5.7–22.3)	10.2 (5.2–19.9)	14.3 (6.6–32.6)	0.026
Age: years (mean ± SD)	55.4 ± 8.6	55.1 ± 8.7	54.1 ± 8.4	0.295
Male (n, %)	295 (84.5)	181 (85)	114 (83.8)	0.890
Cirrhosis (n, %)	314 (90.0)	189 (88.7)	125 (91.9)	0.434
Diabetes mellitus (n, %)	79 (22.6)	51 (23.9)	28 (20.6)	0.549
Hyperlipidemia (n, %)	36 (10.3)	22 (10.3)	14 (10.3)	1.000
Alcohol use (n, %)	16 (4.6)	13 (6.1)	3 (2.2)	0.151
HBV (n, %)	262 (75.1)	163 (76.5)	99 (72.8)	0.510
HBV-medication use before transplant (n, %)	173 (49.6)	104 (48.8)	69 (50.7)	0.812
HCV (n, %)	150 (43.0)	97 (45.5)	53 (39.0)	0.272
HCV-medication use before transplant (n, %)	39 (11.2)	20 (9.4)	19 (14.0)	0.250
Living donor (n, %)	266 (76.2)	163 (76.5)	103 (75.7)	0.968
Transplantation period (n, %)				< 0.001
Before 2008	112 (32.1)	55 (25.8)	57 (41.9)	
2009–2012	134 (38.4)	71 (33.3)	63 (46.3)	
After 2013	103 (29.5)	87 (40.9)	16 (11.8)	

IQR, interquartile range; SD, standard deviation.

### Early Post-Transplant Medications

Immunosuppressive medications majorly used for treatment within 6 months after liver transplantation were tacrolimus (94.3%) and MMF (85.7%). Mammalian target of rapamycin inhibitors (sirolimus and everolimus) was used in 14.0 and 18.6% of patients, respectively (**Table 2**). The distribution of immunosuppressive drug use and their defined daily dose were not different between early and late recurrence groups, except for MMF (171.7 ± 129.5 *vs.* 208.6 ± 159.0, *P* = 0.024) and everolimus (40.2 ± 93.1 *vs.* 21.3 ± 60.4, *P* = 0.022) (**Table S1**).

**Table 2:** Summary of selective medications within 6 months after transplant.

	All recurrence (n = 349)	Recur within 2 years (n = 213)	Recur after 2 years (n = 136)	*P*-value
Immunosuppressants				
Tacrolimus (n, %)	329 (94.3)	198 (93.0)	131 (96.3)	0.279
Cyclosporin (n, %)	24 (6.9)	16 (7.5)	8 (5.9)	0.712
MMF (n, %)	299 (85.7)	178 (83.6)	121 (89.0)	0.212
Sirolimus (n, %)	49 (14.0)	28 (13.1)	21 (15.4)	0.657
Everolimus (n, %)	65 (18.6)	46 (21.6)	19 (14.0)	0.100
Metformin (n, %)	84 (24.1)	51 (23.9)	33 (24.3)	1.000
HBV medication				
Lamivudine (n, %)	80 (22.9)	38 (17.8)	42 (30.9)	0.007
Entecavir (n, %)	102 (29.2)	69 (32.4)	33 (24.3)	0.132
Tenofovir (n, %)	12 (3.4)	9 (4.2)	3 (2.2)	0.479

MMF, mycophenolate mofetil.

Lamivudine was used in 22.9% of patients, and its usage was higher in the late recurrence group than in the early group (30.9 *vs.* 17.8%, *P* = 0.007). Furthermore, lamivudine was mostly (57/80, 71.3%) prescribed in the earlier transplant period (2005–2008): 71.1% in the early recurrence group and 71.4% in the late recurrence group. By contrast, entecavir was prescribed mostly (81/102, 79.4%) in the recent era (2013–2016): 88.4% (61/69) in the early recurrence group and 60.6% (20/33) in the late recurrence group (*P* = 0.003).

### Intervention

Only 20.3% of patients in this cohort received no interventions for HCC within 1 year before transplantation, and the late recurrence group seemed to have more of them than did the early group (25.0 *vs.* 17.4%, *P* = 0.084) (**Table S2**). Within 1 year before transplant surgery, 222 (63.6% in 349) received TACE and estimated 175 (50.1%) at most used TACE as a downstaging strategy (a minimum of 3 months to observe before surgery).

For post-transplant recurrence, the number of patients who received the initial and ever-exposed treatment modalities of resection, RFA, TACE, radiotherapy, and sorafenib was 16, 22, 95, 112, and 71 and 22, 45, 151, 183, and 130, respectively (**Table 3**). Common initial treatments for HCC recurrence were radiotherapy (32.1%), TACE (27.2%), and sorafenib (20.3%). Based on reimbursement regulation for HCC treatment, the initial recurrence HCC stage was estimated to be advanced (vascular or extra-hepatic metastases) for at least 20.3% (71/349) and intrahepatic for at least 38.1% (133/349). Among the 130 (37.2%) patients exposed to sorafenib after recurrence, most (97/130, 74.6%) received other treatments in sequence or in combination. Initial treatment with sorafenib was more common in the early recurrence group than in the late recurrence group (23.0 *vs.* 16.2%, *P* = 0.122), suggesting more advanced HCC, when the first post-transplant recurrence occurred, in the early group. Consistently, sorafenib, as the only single treatment modality throughout the post-recurrence courses, was used more frequently in the early recurrence group than in the late recurrence group (12.7 *vs.* 4.4%, *P* = 0.010).

**Table 3:** Summary of HCC treatment modalities after transplantation in patients with post-transplant recurrence.

	All recurrence (n = 349)	Recur within 2Y (n = 213)	Recur after 2Y (n = 136)	*P*-value
Initial				
Hepatectomy	16 (4.6)	8 (3.8)	8 (5.9)	0.493
RFA	22 (6.3)	12 (5.6)	10 (7.4)	
TACE	95 (27.2)	53 (24.9)	42 (30.9)	
Sorafenib	71 (20.3)	49 (23.0)	22 (16.2)	
RT	112 (32.1)	70 (32.9)	42 (30.9)	
Others	33 (9.5)	21 (9.9)	12 (8.8)	
Ever exposure				
Hepatectomy only	9 (2.6)	3 (1.4)	6 (4.4)	0.024
RFA only	8 (2.3)	4 (1.9)	4 (2.9)	
TACE only	47 (13.5)	27 (12.7)	20 (14.7)	
Sorafenib only	33 (9.5)	27 (12.7)	6 (4.4)	
RT only	69 (19.8)	35 (16.4)	34 (25.0)	
Chemotherapy only	22 (6.3)	13 (6.1)	9 (6.6)	
RFA and TACE	8 (2.3)	5 (2.3)	3 (2.2)	
TACE and RT	27 (7.7)	21 (9.9)	6 (4.4)	
Sorafenib and RT	44 (12.6)	28 (13.1)	16 (11.8)	
TACE, sorafenib, and RT	16 (4.6)	6 (2.8)	10 (7.4)	
Other combinations	66 (18.9)	44 (20.7)	22 (16.2)	

RFA, radiofrequency ablation; RT, radiotherapy; TACE, transarterial chemoembolization.

Data were number (%).

RFA was applied in 45 (12.9%) patients. Nearly half of them (22/45, 48.9%) was initial treatment, and most of them also received other treatments (37/45, 82.2%). The RFA distribution (either initial treatment or treatment exposure) between the early and late recurrence groups was not statistically significant.

Twenty-two (6.3%) patients received resection for post-transplant recurrence in this cohort, and the majority of them received it as the initial treatment (16/22, 72.7%). Over half of the patients (13/22, 59.1%) who received resection also received treatment with other modalities. The number of patients who received only resection was higher in the late recurrence group than in the early recurrence group (4.4 *vs.* 1.4%, *P* = 0.096).

Although radiotherapy is not regarded as the standard treatment for HCC, its application was more common (183, 52.4%) than that of TACE (151/349, 43.3%). Many patients who received radiotherapy (114/183, 62.3%) or TACE (104/151, 68.9%) also received other treatments in their post-recurrence courses.

### Post-Recurrence Survival

**Figure 2:**
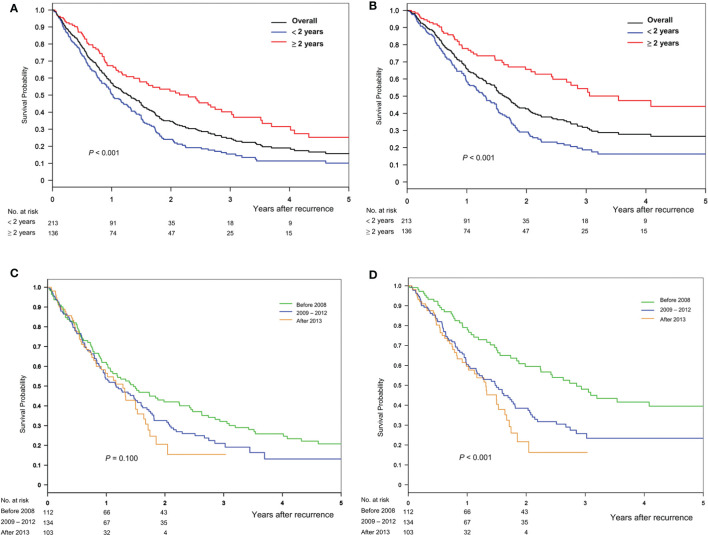
Overall survival and HCC-specific survival after recurrence, stratified based on the timing of **(A, B)** recurrence and **(C, D)** transplant era, respectively.

The median follow-up months after recurrence was 11.2 months (5.7–22.3 months) in the cohort: 10.2 (5.2–19.9) and 14.3 (6.6–32.6) months in the early and late recurrence groups, respectively (*P* = 0.026). The 1-, 2-, 3-, and 4-year post-recurrence overall survival and HCC-specific survival rates were 57.0, 34.7, 24.7, and 19.0 and 66.0, 42.6, 31.9, and 27.8%, respectively. The crude survival periods were higher in the late group than in the early group (for overall, *P* < 0.001; for HCC-specific, *P* < 0.001) (**Figures 2A, B**). The early transplant era (before 2008) showed high survival rates (**Figures 2C, D**).

**Figure 3:**
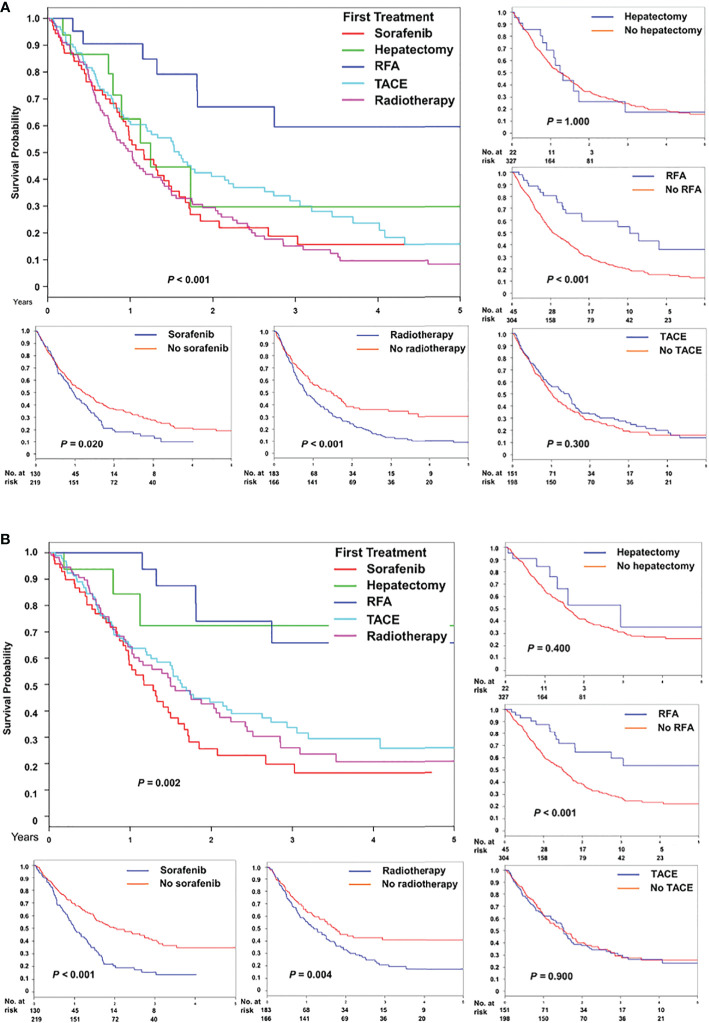
Comparison of **(A)** overall and **(B)** HCC-specific survival after treatment for recurrence, stratified based on five initial modalities, namely sorafenib, hepatectomy, radiofrequency ablation, transarterial chemoembolization, and radiotherapy, and individual modality.

After stratification based on initial treatment modalities for recurrence after liver transplantation, the overall survival was statistically different (*P* < 0.001, **Figure 3A**). A similar pattern was observed for time to HCC-specific death (**Figure 3B**). Furthermore, **Figure 3** presents the overall survival and HCC-specific survival after individual treatment. Superior survival was observed in patients who received RFA, whereas inferior survival was observed in patients who received sorafenib or radiotherapy.

### Univariable and Multivariable Analyses

The results of the univariable analysis suggested that a long interval (>2 years) between transplant and HCC recurrence and initial treatment for recurrence with RFA was significantly associated with better overall survival (**Table S3**). Moreover, these factors were significant in a multivariable analysis, with the adjusted HR of 0.57 (95% CI 0.24–0.77) for recurrence after 2 years and of 0.28 (0.12–0.63) for RFA (compared with sorafenib) (**Table 4**). Compared with the early transplant era (before 2008), while recent periods (2009–2012 and after 2013) suggested a high risk of all-cause mortality in univariable analysis, only transplant period 2009–2012 was significant in multivariable analysis (**Table 4**).

**Table 4:** Prognostic factors for all-cause and HCC mortality after post-transplant recurrence in multivariable analyses.

	All-cause death*	*P*-value	HCC death*	*P*-value
Recur after 2 years	0.57 (0.24–0.77)	<0.001	0.46 (0.32–0.66)	<0.001
Treatment after recurrence				
RFA *vs.* sorafenib	0.28 (0.12–0.63)	0.002	0.24 (0.07–0.81)	0.022
Transplantation periods				
2009–2012 *vs.* before 2008	1.57 (1.03–2.38)	0.036	1.96 (1.19–3.22)	0.008
After 2013 *vs.* before 2008	1.24 (0.73–2.09)	0.426	1.87 (1.01–3.45)	0.047

*Data were adjusted hazard ratios (95% confidence intervals), adjusted for male sex, HBV, HCV, cirrhosis, diabetes, hyperlipidemia, alcohol use, living donor, monthly income, post-transplant medications, and other HCC treatments (hepatectomy, transarterial chemoembolization, radiotherapy, others) after recurrence.

RFA, radiofrequency ablation.

Receiving TACE within 1 year before transplant was observed more frequently in recent era after 2009 than in the early era (*P* = 0.002, **Table S4**). The effect of transplant era was non-significant after stratification by receiving TACE within 1 year before transplant (*P* = 0.124 in patients with prior TACE and *P* = 0.886 in those without prior TACE; **Figure S1**), suggesting the survival difference between the transplant era may be due to more frequent use of TACE as downstaging tools recently.

Consistently, the results of the multivariable analysis showed that recurrence after 2 years was associated with a long survival from HCC recurrence to HCC death (**Table S5**), and initial treatment with RFA was associated with a low risk of cancer death. Compared with the early transplant era (before 2008), recent periods (2009–2012 and after 2013) were associated with a high risk of cancer death in both univariable and multivariable analyses.

HCV, alcohol use, and everolimus were associated with a high risk of all-cause and cancer death based on a univariable analysis but not significantly according to a multivariable analysis. Living donor appeared as a risk factor in a univariable analysis for cancer death with an HR 1.50 (1.04–2.15) but not significant in a multivariable analysis and not for all-cause death. Further sensitivity analysis with stringent donor assignment criteria showed consistent results (**Tables S6** and **S7**).

### Subgroup Analysis

**Figure 4:**
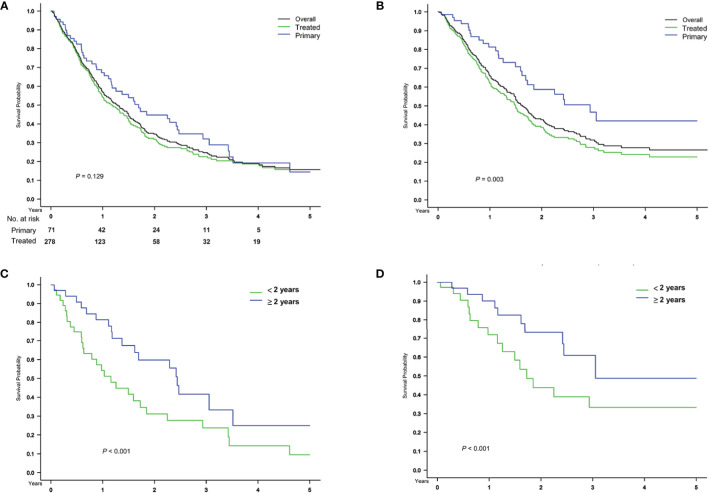
Subgroup analysis in patients with primary HCC treated by upfront transplant. Comparison of **(A)** overall and **(B)** HCC-specific survival after treatment for recurrence, stratified based on primary and treated HCC before transplant surgery and on the timing of **(C, D)** recurrence in 71 patients with primary HCC treated by upfront transplant.

A total of 71 patients with primary HCC treated by upfront transplant were identified. The median follow-up month after recurrence was 15.1 months (7.1–29.5 months). Compared to the previously treated patients (**Figure 4A**, green curve), these patients (**Figure 4A**, blue curve) had similar post-recurrence overall survival but superior HCC-specific survival (**Figure 4B**, *P* = 0.003). In this subgroup, the HCC cancer stage met the UCSF criteria (without potentially confounded by heterogeneous original tumor status and previous treatment effects), and the survival difference between early and late recurrence was consistent with the findings on overall study population, either all-cause or HCC-related death (**Figures 4C, D**, *P* < 0.001).

## Discussion

Our study revealed four main findings. First, the post-recurrence survival of liver transplant recipients was time-dependent; early recurrence after liver transplant and recent transplant era were two independent risk factors for an inferior outcome. The effect of transplant era was associated with recently receiving pretransplant TACE. Second, the majority of patients (79.7%) received HCC treatments within 1 year before transplant, and TACE was the commonest application, implying a large percent of salvage liver transplant in this cohort. Third, the most common initial treatment for post-transplant recurrence was radiotherapy (32.1%), followed by TACE (27.2%) and sorafenib (20.3%). Over half of the cohort (54.0%) received only one treatment after recurrence. Lastly, when recurrence cancer stage allowed eligible interventions, RFA was associated with a superior outcome, whereas radiotherapy and sorafenib use was associated with an inferior outcome.

It is well known that disease stage is closely related to prognosis in cancer patients, and the characteristics of the transplant recipients are quite heterogeneous. Hong et al. showed, in a large South Korean single center study of 92 patients, that HCC size >5 cm at explants was associated with poor post-recurrence survival in recurrence within 6 months after transplant surgery but did not remain significant in recurrence that occurred 6 months later (16). Bodzin et al. showed that, in a largest US single center study of 106 patients, post-transplant HCC recurrence stage, rather than the primary or at transplant HCC stage, predicted post-recurrence mortality (14). The overall information for the initial primary HCC stage and definite HCC-recurrence disease stage of the patients was not available in Taiwan NHIRD. However, the heterogeneous combinations of tumor status at different stages (primary, at transplant, and post-transplant recurrence) in our large cohort would probably make the results toward the null. Moreover, our study showed the effect of recurrence timing on post-recurrence survival in a pure subgroup of patients with primary HCC treated with upfront transplant was consistent with that of the main cohort. The effect of recurrence timing on post-recurrence survival might be a robust conclusion.

The median time between transplant to HCC recurrence in our cohort, the largest one in the literature to our knowledge, was 17.8 months (IQR, 10.7–34.4 months), and 61.0% patients had recurrence within 2 years of transplantation. Consistently, peak HCC recurrence occurs within 2–3 years after transplant (3, 25, 26), and early HCC recurrence portends the worst prognosis (3, 27–29). Furthermore, a longer survival was observed in late than in early recurrence after liver resection for HCC (30). Verna et al. suggested different plausible biological mechanisms, explaining early and late post-transplant recurrence (3). Early recurrence could be due to non-detected extrahepatic metastases that may be present before transplant and as a consequence of circulating HCC clones engrafting and growing in a target organ after liver transplantation (3). Late recurrence could be due to a second unknown hit that may lead to late engrafting of HCC cells that are less in number and remained latent for a long time during the post-transplant period (3). Our data supports the statement of intense surveillance during the first 2 years after transplant (3) and justifies the urgent need for effective adjuvant therapy in this critical period. It is noteworthy that the use of mammalian target of rapamycin inhibitors early (within 6 months after transplant) in the post-transplant period did not show survival benefit in our cohort. Nonetheless, we provided the benchmark reference for future trial design and historical comparison.

HCC treatments before liver transplant can be due to several reasons: to meet transplantable criteria (downstaging tumor status), to bridge (extending waiting time), to salvage (treating transplantable HCC recurrence after other treatments), or to treat liver decompensation (non-tumor liver factor). For example, over half of our main cohort received TACE within 1 year before transplant surgery. We could not tell apart exactly the rationales of TACE treatment based solely on current databases. However, the goal of all these efforts before transplant is to increase the transplantable rates and to reduce wait-list dropout.

RFA appeared as a modality with superior comparative effectiveness in multiple dimensions of our analysis. RFA showed a comparative survival benefit in treating intrahepatic recurrence after liver resection in our previous hospital cohort (21) and another recent report (31). Because extrahepatic metastasis was observed more often in post-transplant than in postresection recurrence (1), the bias in selecting HCC-recurrent patients who were eligible for RFA is estimated to be more in the heterogeneous transplant setting. However, data on liver-directed therapy for the treatment of post-transplant HCC recurrence are lacking and limited to small case series (3). Our results contribute to the literature on the feasibility of RFA treatment for intrahepatic recurrence, which was the best determinant for the prognosis.

Initial treatment with sorafenib and its ever exposure in 20.3 and 37.2% of this cohort patients, respectively, composed a recurrence subgroup of a particular advanced stage, according to reimbursement criteria. The overall 1-year survival rate with initial treatment with sorafenib was 43.7% in our study, which was a bit lower than a pooled estimate at 63% in a meta-analysis (32). The widespread use of radiotherapy in the initial treatment for post-transplant HCC recurrence suggested bone metastasis, which is one of the most common extrahepatic sites. However, the survival benefit of radiotherapy in our study was limited. All of these highlight the therapeutic gap and warrant an investigator-initiated trial to tackle this problem.

This study is limited by the built-in shortage of no information of laboratory data (such as alpha-fetoprotein), and radiographic and pathological findings regarding tumor status in the claim database, which impedes the risk factor analysis between recurrence and non-recurrence. This might influence the analysis of post-recurrence survival. Some received pre-transplant TACE or other specific treatments probably due to the preference of transplant surgeon. This might bias the overall analysis. However, only by utilizing this large multicenter cohort, we could possibly dilute the potential bias, demonstrate the trend of the real-world nature of this heterogeneous cohort, and pave the road for tailoring potential therapeutic implications into future practice. Additionally, resection was not popular in Taiwan, and limited number of patients (n = 22) precluded a balanced assessment, although resection seems to improve cancer-specific survival.

## Conclusion

In Taiwan, management of HCC recurrence after liver transplantation was heterogeneous. Patients with HCC recurrence within 2 years after liver transplantation had the highest mortality risk. This subgroup cohort is ideal for future interventional trial design. Our data further support the statement of intense surveillance during the early period (first 2 years) after transplant and justify the urgent need for effective adjuvant treatments in this critical period.

## Data Availability Statement

The raw data supporting the conclusions of this article will be made available by the authors, without undue reservation.

## Ethics Statement

The studies involving human participants were reviewed and approved by the Institutional Review Board of National Taiwan University Hospital. Written informed consent for participation was not required for this study in accordance with the national legislation and the institutional requirements.

## Author Contributions

CMH and JYW made the concept and design. CMH, JYW, JFZ, and CHL acquired, analyzed, or interpreted the data. CMH drafted the manuscript. CMH, JYW, and CHC critically revised the manuscript for important intellectual content. JFZ and CHC conducted the statistical analysis. JYW obtained the funding. CHC provided administrative, technical, or material support. RHH and PHL supervised the study. All authors contributed to the article and approved the submitted version.

## Funding

This study was funded by the Ministry of Health and Welfare (MOHW109-CDC-C-114-000108). The funder played no role in the study design, data analysis, or manuscript drafting.

## Conflict of Interest

The authors declare that the research was conducted in the absence of any commercial or financial relationships that could be construed as a potential conflict of interest.

## Supplementary Material

Supplementary Figure 1 Overall survival after recurrence in patients receiving **(A)** and not receiving **(B)** transarterial chemoembolization (TACE) within 1 year before transplant in three transplant eras.

Supplementary Table 1 Defined daily dose of selective medications within 6 months after transplant.

Supplementary Table 2 Summary of HCC treatment modalities before transplantation in patients with post-transplant recurrence.

Supplementary Table 3 Prognostic factors for all-cause mortality after post-transplant recurrence.

Supplementary Table 4 Receiving transarterial chemoembolization (TACE) for hepatocellular carcinoma within one year before liver transplantation during three transplant era.

Supplementary Table 5 Prognostic factors for HCC mortality after post-transplant recurrence.

Supplementary Table 6 Prognostic factors for all-cause mortality after HCC recurrence in a sensitivity analysis of stringent donor allocation.

Supplementary Table 7 Prognostic factors for HCC mortality after post-transplant recurrence in a sensitivity analysis of stringent donor allocation.

## Abbreviations

ATC, anatomical therapeutic chemical; DDD, defined daily dose; ICD, International Classification of Diseases; IQR, inter-quartile range; MMF, mycophenolate mofetil; mTOR, mammalian target of rapamycin; NHIRD, National Health Insurance Research Database; RCIPD, Registry for Catastrophic Illness Patient Database; RFA, radiofrequency ablation; TACE, transarterial chemoembolization.
