# Supplementary figures and images for: Survival After Treatable Hepatocellular Carcinoma Recurrence in Liver Recipients: A Nationwide Cohort Analysis
Source: Front Oncol. 2021 Jan 28;10:616094. doi: 10.3389/fonc.2020.616094 (PMC7883828; doi:10.3389/fonc.2020.616094)

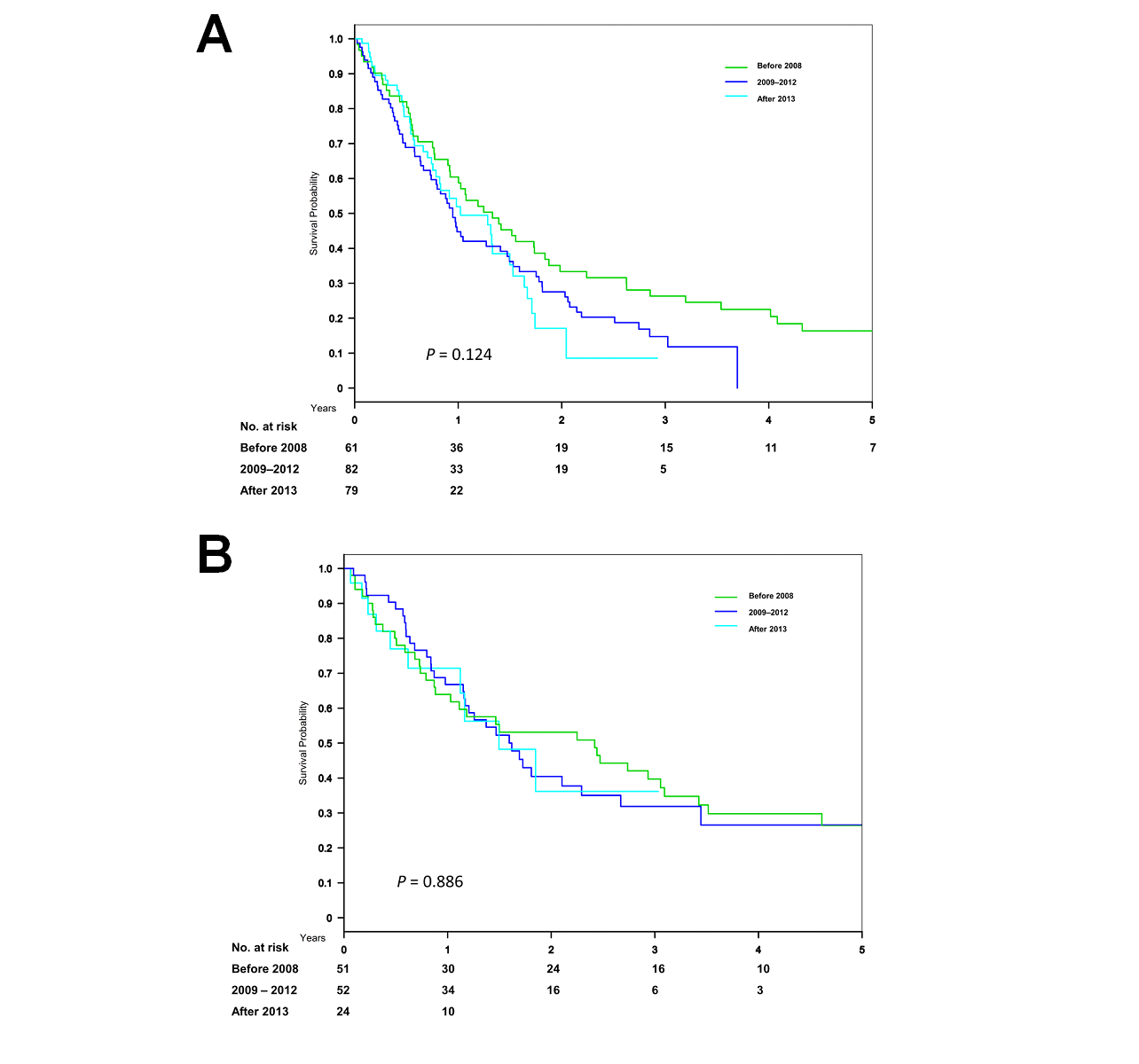

Supplement: Supplementary Figure 1 — Overall survival after recurrence in patients receiving (A) and not receiving (B) transarterial chemoembolization (TACE) within 1 year before transplant in three transplant eras. [file Image_1.tiff]
