# Supplementary material for: Survival After Treatable Hepatocellular Carcinoma Recurrence in Liver Recipients: A Nationwide Cohort Analysis
Source: Front Oncol. 2021 Jan 28;10:616094. doi: 10.3389/fonc.2020.616094 (PMC7883828; doi:10.3389/fonc.2020.616094)
Supplement: Supplementary file 2 [file DataSheet_1.docx]

**Supplemental methods**

**Procedure code**

| **Intervention** | **Procedure code** |
| --- | --- |
| Hepatectomy | 75002B, 75003B, 75004B, 75005B, 75015B, 75016B, 75017B, 75018B |
| Radiofrequency ablation | 37042C, 37043C, 37044C |
| Transarterial chemoembolization | 33075B |
| Radiotherapy | 36001B, 36004B, 36005B, 36006B, 36009B, 36010B, 36011B, 36012B, 36013B, 36015B, 36020B, 37013B, 39019B |
| Sorafenib | BC24727100, B024727100 |
| Chemotherapy | 37038B, 37029B, 37040B |
| Alcohol injection | 39020B |

**International Classification of Disease-9 Clinical Modification (ICD-9-CM) code**

| **Disease** | **ICD-9-CM code** |
| --- | --- |
| Diagnosis of hepatitis B virus | 070.2, 070.3 |
| Diagnosis of hepatitis C virus | 070.41, 070.44, 070.51, 070.54, 070.7 |

**Anatomical Therapeutic Chemical (ATC) classification code**

| **Drug for** | **ATC code** |
| --- | --- |
| Hepatitis B virus | J05AF05, J05AF07, J05AF08, J05AF10, J05AF11, J05AF13 |
| Hepatitis C virus | L03AB, J05AP |
