# Supplementary material for: Survival After Treatable Hepatocellular Carcinoma Recurrence in Liver Recipients: A Nationwide Cohort Analysis
Source: Front Oncol. 2021 Jan 28;10:616094. doi: 10.3389/fonc.2020.616094 (PMC7883828; doi:10.3389/fonc.2020.616094)
Supplement: Supplementary Table 1 — Defined daily dose of selective medications within 6 months after transplant. [file Table_1.docx]

**Table S1.** Defined daily dose of selective medications within 6 months after transplant

|  | All recurrence  (n = 349) | Recur within 2 years  (n = 213) | Recur after 2 years  (n = 136) | *P*-value |
| --- | --- | --- | --- | --- |
| Immunosuppressants |  |  |  |  |
| Tacrolimus (mean ± SD) | 387.2 ± 286.4 | 368.5 ± 286.2 | 416.6 ± 285.2 | 0.125 |
| Cyclosporin (mean ± SD) | 21.6 ± 92.3 | 23.9 ± 100.4 | 18.3 ± 78.2 | 0.545 |
| MMF (mean ± SD) | 186.1 ± 142.7 | 171.7 ± 129.5 | 208.6 ± 159.0 | 0.024 |
| Sirolimus (mean ± SD) | 27.1 ± 79.3 | 28.1 ± 84.0 | 25.4 ± 71.7 | 0.754 |
| Everolimus (mean ± SD) | 32.8 ± 82.4 | 40.2 ± 93.1 | 21.3 ± 60.4 | 0.022 |
| Metformin (mean ± SD) | 14.3 ± 37.4 | 13.6 ± 36.3 | 15.4 ± 39.0 | 0.660 |
| HBV medication |  |  |  |  |
| Lamivudin (mean ± SD) | 50.6 ± 102.0 | 36.1 ± 85.6 | 73.4 ± 120.1 | 0.002 |
| Entecavir (mean ± SD) | 67.8 ± 124.1 | 76.6 ± 131.2 | 54.1 ± 111.1 | 0.086 |
| Tenofovir (mean ± SD) | 6.0 ± 34.8 | 7.5 ± 38.8 | 3.7 ± 27.4 | 0.289 |

MMF, mycophenolate mofetil
