# Supplementary material for: Survival After Treatable Hepatocellular Carcinoma Recurrence in Liver Recipients: A Nationwide Cohort Analysis
Source: Front Oncol. 2021 Jan 28;10:616094. doi: 10.3389/fonc.2020.616094 (PMC7883828; doi:10.3389/fonc.2020.616094)
Supplement: Supplementary Table 2 — Summary of HCC treatment modalities before transplantation in patients with post-transplant recurrence. [file Table_2.docx]

**Table S2.** Summary of HCC treatment modalities before transplantation in patients with post-transplant recurrence

|  | All recurrence  (n = 349) | Recur within 2 years  (n = 213) | Recur after 2 years  (n = 136) | *P*-value |
| --- | --- | --- | --- | --- |
| Within 1 year before transplant (n, %) |  |  |  | 0.165 |
| None | 71 (20.3) | 37 (17.4) | 34 (25.0) |  |
| Hepatectomy only | 10 (2.9) | 6 (2.8) | 4 (2.9) |  |
| RFA only | 20 (5.7) | 10 (4.7) | 10 (7.4) |  |
| PTA only | 9 (2.6) | 4 (1.9) | 5 (3.7) |  |
| TACE only | 134 (38.4) | 90 (42.3) | 44 (32.4) |  |
| RFA and TACE | 36 (10.3) | 23 (10.8) | 13 (9.6) |  |
| PTA and TACE | 11 (3.2) | 4 (1.9) | 7 (5.1) |  |
| Others | 58 (16.6) | 39 (18.3) | 19 (14.0) |  |

PTA, percutaneous alcohol injection; RFA, radiofrequency ablation; RT, radiotherapy; TACE, transarterial chemoembolization
