# Supplementary material for: Survival After Treatable Hepatocellular Carcinoma Recurrence in Liver Recipients: A Nationwide Cohort Analysis
Source: Front Oncol. 2021 Jan 28;10:616094. doi: 10.3389/fonc.2020.616094 (PMC7883828; doi:10.3389/fonc.2020.616094)
Supplement: Supplementary Table 3 — Prognostic factors for all-cause mortality after post-transplant recurrence. [file Table_3.docx]

**Table S3.** Prognostic factors for all-cause mortality after post-transplant recurrence

|  | Crude HR (95%CI) | *P*-value | Adjusted HR* (95%CI) | *P*-value |
| --- | --- | --- | --- | --- |
| Recur after 2 years | 0.53 (0.40-0.70) | < 0.001 | 0.57 (0.24-0.77) | < 0.001 |
| HBV | 0.93 (0.69-1.24) | 0.604 | 1.05 (0.72-1.62) | 0.705 |
| HCV | 1.35 (1.04-1.75) | 0.025 | 1.17 (0.86-1.59) | 0.311 |
| Cirrhosis | 1.02 (0.67-1.56) | 0.921 | 1.10 (0.70-1.71) | 0.689 |
| Diabetes | 1.19 (0.87-1.63) | 0.274 | 1.08 (0.72-1.62) | 0.705 |
| Alcohol use | 1.61 (0.91-2.82) | 0.099 | 0.97 (0.51-1.86) | 0.937 |
| Living donor | 1.11 (0.83-1.48) | 0.492 | 0.94 (0.65-1.35) | 0.724 |
| Monthly income (TWD) |  |  |  |  |
| 16500–26400 *vs*. < 16500 | 1.45 (0.96-2.19) | 0.077 | 1.33 (0.86-2.05) | 0.198 |
| > 26400 *vs*. < 16500 | 1.31 (0.88-1.94) | 0.183 | 1.17 (0.78-1.77) | 0.448 |
| Post-transplant medications |  |  |  |  |
| Tacrolimus | 1.52 (0.85-2.72) | 0.157 | 1.23 (0.59-2.53) | 0.582 |
| Cyclosporin | 0.92 (0.56-1.51) | 0.747 | 1.04 (0.61-1.76) | 0.893 |
| MMF | 1.24 (0.84-1.83) | 0.281 | 1.20 (0.73-1.97) | 0.463 |
| Sirolimus | 0.94 (0.66-1.33) | 0.721 | 0.81 (0.54-1.20) | 0.290 |
| Everolimus | 1.38 (0.95-2.02) | 0.092 | 0.98 (0.62-1.56) | 0.941 |
| Metformin | 1.16 (0.86-1.58) | 0.326 | 1.09 (0.75-1.58) | 0.650 |
| Lamivudine | 0.74 (0.53-1.04) | 0.087 | 0.94(0.63-1.39) | 0.750 |
| Entecavir | 1.10 (0.79-1.54) | 0.577 | 0.82(0.56-1.21) | 0.320 |
| Treatment after recurrence |  |  |  |  |
| Hepatectomy *vs.* sorafenib | 1.26 (0.62-2.58) | 0.522 | 0.86 (0.40-1.84) | 0.694 |
| RFA *vs.* sorafenib | 0.33 (0.12-0.90) | 0.030 | 0.28 (0.12-0.63) | 0.002 |
| TACE *vs.* sorafenib | 0.93 (0.46-1.88) | 0.850 | 0.79 (0.52-1.21) | 0.279 |
| RT *vs.* sorafenib | 1.37 (0.69-2.71) | 0.372 | 1.18 (0.78-1.78) | 0.424 |
| Others *vs.* sorafenib | 1.24 (0.56-2.72) | 0.597 | 1.03 (0.59-1.80) | 0.917 |
| Transplantation period |  |  |  |  |
| 2009–2012 *vs*. before 2008 | 1.30 (0.97-1.76) | 0.081 | 1.57 (1.03-2.38) | 0.036 |
| After 2013 *vs*. before 2008 | 1.42 (0.98-2.03) | 0.061 | 1.24 (0.73-2.09) | 0.426 |

*Adjusted for male sex and hyperlipidemia

MMF, mycophenolate mofetil; RFA, radiofrequency ablation; RT, radiotherapy; TACE, transarterial chemoembolization
