# Supplementary material for: Survival After Treatable Hepatocellular Carcinoma Recurrence in Liver Recipients: A Nationwide Cohort Analysis
Source: Front Oncol. 2021 Jan 28;10:616094. doi: 10.3389/fonc.2020.616094 (PMC7883828; doi:10.3389/fonc.2020.616094)
Supplement: Supplementary Table 4 — Receiving transarterial chemoembolization (TACE) for hepatocellular carcinoma within one year before liver transplantation during three transplant era. [file Table_4.docx]

**Table S4.** Receiving transarterial chemoembolization (TACE) for hepatocellular carcinoma within one year before liver transplantation during three transplant era

| (n, %) | Receiving TACE | Not receiving TACE | *P* |
| --- | --- | --- | --- |
| Before 2008 | 61 (27.5) | 51 (40.2) | 0.002 |
| 2009–2012 | 82 (36.9) | 52 (40.9) |  |
| After 2013 | 79 (35.6) | 24 (18.9) |  |
