# Supplementary material for: Survival After Treatable Hepatocellular Carcinoma Recurrence in Liver Recipients: A Nationwide Cohort Analysis
Source: Front Oncol. 2021 Jan 28;10:616094. doi: 10.3389/fonc.2020.616094 (PMC7883828; doi:10.3389/fonc.2020.616094)
Supplement: Supplementary Table 5 — Prognostic factors for HCC mortality after post-transplant recurrence. [file Table_5.docx]

**Table S5.** Prognostic factors for HCC mortality after post-transplant recurrence

|  | Crude HR (95%CI) | *P*-value | Adjusted HR* (95%CI) | *P*-value |
| --- | --- | --- | --- | --- |
| Recur after 2 years | 0.41 (0.29-0.58) | < 0.001 | 0.46 (0.32-0.66) | < 0.001 |
| HBV | 1.00 (0.71-1.41) | 0.998 | 1.01 (0.66-1.56) | 0.962 |
| HCV | 1.42 (1.06-1.92) | 0.020 | 1.11 (0.78-1.58) | 0.549 |
| Cirrhosis | 0.93 (0.58-1.48) | 0.760 | 1.06 (0.64-1.74) | 0.829 |
| Diabetes | 1.17 (0.81-1.69) | 0.392 | 1.12 (0.70-1.79) | 0.649 |
| Alcohol use | 1.99 (1.11-3.60) | 0.022 | 1.02 (0.51-2.03) | 0.973 |
| Living donor | 1.50 (1.04-2.15) | 0.028 | 1.06 (0.67-1.67) | 0.805 |
| Monthly income (TWD) |  |  |  |  |
| 16500–26400 *vs*. < 16500 | 1.43 (0.89-2.31) | 0.142 | 1.16 (0.70-1.92) | 0.556 |
| > 26400 *vs*. < 16500 | 1.35 (0.86-2.14) | 0.196 | 1.15 (0.72-1.85) | 0.554 |
| Post-transplant medications |  |  |  |  |
| Tacrolimus | 1.26 (0.68-2.32) | 0.460 | 0.84 (0.37-1.91) | 0.681 |
| Cyclosporin | 1.00 (0.58-1.73) | 0.997 | 1.20 (0.66-2.16) | 0.552 |
| MMF | 1.55 (0.95-2.53) | 0.077 | 1.76 (0.92-3.37) | 0.089 |
| Sirolimus | 0.95 (0.64-1.42) | 0.811 | 0.84 (0.53-1.31) | 0.435 |
| Everolimus | 1.80 (1.20-2.70) | 0.004 | 1.08 (0.66-1.78) | 0.756 |
| Metformin | 1.08 (0.75-1.54) | 0.683 | 1.02 (0.65-1.58) | 0.947 |
| Lamivudine | 0.67 (0.45-1.01) | 0.057 | 0.93 (0.58-1.48) | 0.764 |
| Entecavir | 1.30 (0.89-1.90) | 0.181 | 0.83 (0.54-1.28) | 0.396 |
| Treatment after recurrence |  |  |  |  |
| Hepatectomy *vs.* sorafenib | 0.38 (0.14-1.06) | 0.064 | 0.53 (0.15-1.90) | 0.327 |
| RFA *vs.* sorafenib | 0.52 (0.14-1.95) | 0.335 | 0.24 (0.07-0.81) | 0.022 |
| TACE *vs.* sorafenib | 0.56 (0.20-1.56) | 0.266 | 0.85 (0.50-1.44) | 0.546 |
| RT *vs.* sorafenib | 0.49 (0.18-1.35) | 0.169 | 1.10 (0.65-1.87) | 0.711 |
| Others *vs.* sorafenib | 0.60 (0.19-1.85) | 0.372 | 0.79 (0.38-1.65) | 0.536 |
| Transplantation periods |  |  |  |  |
| 2009–2012 *vs*. before 2008 | 1.85 (1.29-2.65) | < 0.001 | 1.96 (1.19-3.22) | 0.008 |
| After 2013 *vs*. before 2008 | 2.36 (1.55-3.59) | < 0.001 | 1.87 (1.01-3.45) | 0.047 |

*Adjusted for male sex and hyperlipidemia

MMF, mycophenolate mofetil; RFA, radiofrequency ablation; RT, radiotherapy; TACE, transarterial chemoembolization
