# Supplementary material for: Survival After Treatable Hepatocellular Carcinoma Recurrence in Liver Recipients: A Nationwide Cohort Analysis
Source: Front Oncol. 2021 Jan 28;10:616094. doi: 10.3389/fonc.2020.616094 (PMC7883828; doi:10.3389/fonc.2020.616094)
Supplement: Supplementary Table 6 — Prognostic factors for all-cause mortality after HCC recurrence in a sensitivity analysis of stringent donor allocation. [file Table_6.docx]

**Table S6.** Prognostic factors for all-cause mortality after HCC recurrence in a sensitivity analysis of stringent donor allocation

|  | Crude HR (95%CI) | *P*-value | Adjusted HR* (95%CI) | *P*-value |
| --- | --- | --- | --- | --- |
| Recur after 2 years | 0.66 (0.48-0.90) | 0.009 | 0.78 (0.55-1.09) | 0.148 |
| HBV | 0.85 (0.62-1.17) | 0.324 | 0.90 (0.59-1.37) | 0.623 |
| HCV | 1.48 (1.10-1.98) | 0.009 | 1.40 (0.98-2.00) | 0.068 |
| Cirrhosis | 1.04 (0.65-1.67) | 0.868 | 1.16 (0.69-1.94) | 0.570 |
| Diabetes | 1.26 (0.88-1.78) | 0.205 | 1.00 (0.61-1.64) | 0.999 |
| Alcohol use | 1.30 (0.64-2.65) | 0.468 | 0.74 (0.33-1.64) | 0.455 |
| Living donor | 1.24 (0.91-1.68) | 0.175 | 0.91 (0.62-1.35) | 0.648 |
| Monthly income (TWD) |  |  |  |  |
| 16500–26400 *vs*. < 16500 | 1.68 (1.05-2.70) | 0.030 | 1.46 (0.89-2.40) | 0.135 |
| > 26400 *vs.* < 16500 | 1.57 (1.01-2.43) | 0.045 | 1.40 (0.89-2.21) | 0.143 |
| Post-transplant medications |  |  |  |  |
| Tacrolimus | 1.72 (0.88-3.37) | 0.112 | 1.29 (0.55-2.98) | 0.559 |
| Cyclosporin | 0.86 (0.51-1.46) | 0.579 | 0.94 (0.52-1.68) | 0.825 |
| MMF | 1.19 (0.77-1.85) | 0.428 | 1.10 (0.63-1.91) | 0.737 |
| Sirolimus | 1.11 (0.74-1.67) | 0.614 | 0.98 (0.62-1.54) | 0.916 |
| Everolimus | 1.42 (0.93-2.17) | 0.104 | 1.02 (0.61-1.73) | 0.931 |
| Metformin | 1.36 (0.96-1.91) | 0.082 | 1.10 (0.63-1.91) | 0.737 |
| Lamivudine | 0.74 (0.55-1.00) | 0.051 | 1.10 (0.69-1.76) | 0.689 |
| Entecavir | 0.98 (0.72-1.31) | 0.868 | 1.01 (0.64-1.61) | 0.962 |
| Treatment after recurrence |  |  |  |  |
| Hepatectomy *vs.* sorafenib | 1.11 (0.49-2.51) | 0.798 | 1.28 (0.51-3.24) | 0.600 |
| RFA *vs.* sorafenib | 0.29 (0.11-0.75) | 0.011 | 0.32 (0.12-0.86) | 0.024 |
| TACE *vs.* sorafenib | 0.80 (0.52-1.25) | 0.326 | 0.83 (0.51-1.35) | 0.452 |
| RT *vs.* sorafenib | 1.19 (0.79-1.79) | 0.409 | 1.46 (0.91-2.35) | 0.115 |
| Others *vs.* sorafenib | 1.17 (0.67-2.06) | 0.582 | 1.31 (0.72-2.39) | 0.379 |
| Periods |  |  |  |  |
| 2009–2012 *vs*. before 2008 | 1.35 (0.97-1.88) | 0.075 | 1.70 (1.06-2.73) | 0.028 |
| After 2013 *vs*. before 2008 | 1.63 (1.08-2.45) | 0.019 | 1.71 (0.95-3.07) | 0.073 |

*Adjusted for male sex and hyperlipidemia

MMF, mycophenolate mofetil; RFA, radiofrequency ablation; RT, radiotherapy; TACE, transarterial chemoembolization
