# Supplementary material for: Survival After Treatable Hepatocellular Carcinoma Recurrence in Liver Recipients: A Nationwide Cohort Analysis
Source: Front Oncol. 2021 Jan 28;10:616094. doi: 10.3389/fonc.2020.616094 (PMC7883828; doi:10.3389/fonc.2020.616094)
Supplement: Supplementary Table 7 — Prognostic factors for HCC mortality after post-transplant recurrence in a sensitivity analysis of stringent donor allocation. [file Table_7.docx]

**Table S7.** Prognostic factors for HCC mortality after post-transplant recurrence in a sensitivity analysis of stringent donor allocation

|  | Crude HR (95%CI) | *P*-value | Adjusted HR* (95%CI) | *P*-value |
| --- | --- | --- | --- | --- |
| Recur after 2 years | 0.53 (0.36-0.77) | < 0.001 | 0.65 (0.42-0.99) | 0.045 |
| HBV | 0.96 (0.65-1.41) | 0.839 | 0.89 (0.54-1.46) | 0.640 |
| HCV | 1.53 (1.09-2.15) | 0.015 | 1.25 (0.83-1.89) | 0.288 |
| Cirrhosis | 0.96 (0.56-1.64) | 0.874 | 1.21 (0.67-2.18) | 0.538 |
| Diabetes | 1.32 (0.88-1.98) | 0.181 | 1.21 (0.68-2.14) | 0.522 |
| Alcohol use | 1.57 (0.73-3.36) | 0.247 | 0.69 (0.29-1.66) | 0.405 |
| Living donor | 1.66 (1.14-2.43) | 0.008 | 1.01 (0.63-1.62) | 0.970 |
| Monthly income (TWD) |  |  |  |  |
| 16500–26400 *vs*. < 16500 | 1.61 (0.96-2.69) | 0.071 | 1.29 (0.72-2.32) | 0.395 |
| > 26400 *vs*. < 16500 | 1.65 (0.95-2.88) | 0.076 | 1.40 (0.82-2.38) | 0.220 |
| Post-transplant medications |  |  |  |  |
| Tacrolimus | 1.26 (0.64-2.48) | 0.507 | 0.80 (0.32-2.02) | 0.643 |
| Cyclosporin | 0.94 (0.52-1.70) | 0.832 | 0.99 (0.51-1.91) | 0.977 |
| MMF | 1.47 (0.85-2.56) | 0.172 | 1.54 (0.73-3.26) | 0.259 |
| Sirolimus | 1.11 (0.69-1.79) | 0.666 | 0.88 (0.52-1.51) | 0.648 |
| Everolimus | 1.86 (1.18-2.95) | 0.008 | 1.14 (0.63-2.04) | 0.666 |
| Metformin | 1.29 (0.86-1.94) | 0.218 | 0.97 (0.56-1.70) | 0.926 |
| Lamivudine | 0.63 (0.44-0.90) | 0.012 | 1.07 (0.61-1.87) | 0.810 |
| Entecavir | 1.15 (0.83-1.60) | 0.390 | 0.92 (0.55-1.55) | 0.748 |
| Treatment after recurrence |  |  |  |  |
| Hepatectomy *vs.* sorafenib | 0.53 (0.16-1.74) | 0.296 | 0.76 (0.21-2.78) | 0.677 |
| RFA *vs.* sorafenib | 0.19 (0.06-0.62) | 0.006 | 0.22 (0.06-0.74) | 0.015 |
| TACE *vs.* sorafenib | 0.76 (0.48-1.22) | 0.256 | 0.92 (0.54-1.57) | 0.769 |
| RT *vs.* sorafenib | 0.83 (0.52-1.31) | 0.419 | 1.26 (0.74-2.12) | 0.396 |
| Others *vs.* sorafenib | 0.75 (0.38-1.50) | 0.421 | 0.89 (0.43-1.85) | 0.749 |
| Periods |  |  |  |  |
| 2009–2012 *vs*. before 2008 | 1.93 (1.29-2.88) | 0.001 | 2.20 (1.25-3.87) | 0.006 |
| After 2013 *vs*. before 2008 | 2.70 (1.69-4.34) | <0.001 | 2.65 (1.32-5.31) | 0.006 |

*Adjusted for male sex and hyperlipidemia

MMF, mycophenolate mofetil; RFA, radiofrequency ablation; RT, radiotherapy; TACE, transarterial chemoembolization
